# LAG3 and its emerging role in cancer immunotherapy

**DOI:** 10.1002/ctm2.365

**Published:** 2021-03-24

**Authors:** Miao Wang, Qi Du, Jiangtao Jin, Yuhan Wei, Yuting Lu, Qin Li

**Affiliations:** ^1^ Department of Oncology, Beijing Friendship Hospital Capital Medical University Beijing China; ^2^ Department of Intervention Therapy Zezhou People's Hospital Jincheng Shanxi Province China

**Abbreviations**
ACC: adrenocortical carcinoma
BLCA: bladder urothelial carcinoma
BRCA: breast invasive carcinoma
CESC: cervical squamous cell carcinoma and endocervical adenocarcinoma
CHOL: cholangiocarcinoma
COAD: colon adenocarcinoma
CTLA4: cytotoxic T‐lymphocyte associated protein 4
DLBC: lymphoid neoplasm diffuse large B‐cell lymphoma
ESCA: esophageal carcinoma
GBM: glioblastoma multiforme
GEPIA: Gene Expression Profiling Interactive Analysis
HNSC: head and neck squamous cell carcinoma
ICIs: immune checkpoint inhibitors
KICH: kidney chromophobe
KIRC: kidney renal clear cell carcinoma
KIRP: kidney renal papillary cell carcinoma
LAG3: lymphocyte‐associated gene 3
LAML: acute myeloid leukemia
LGG: brain lower grade glioma
LIHC: liver hepatocellular carcinoma
LUAD: lung adenocarcinoma
LUSC: lung squamous cell carcinoma
MESO: mesothelioma
MHC II: major histocompatibility complex II
OV: ovarian serous cystadenocarcinoma
PAAD: pancreatic adenocarcinoma
PCPG: pheochromocytoma and paraganglioma
PD1: programmed cell death 1
PDL1: programmed cell death ligand 1
PRAD: prostate adenocarcinoma
READ: rectum adenocarcinoma
SARC: sarcoma
SKCM: skin cutaneous melanoma
sLAG3: soluble LAG3
STAD: stomach adenocarcinoma
STES: stomach and esophageal carcinoma
TCGA: The Cancer Genome Atlas
TGCT: testicular germ cell tumors
THCA: thyroid carcinoma
THYM: thymoma
TIL: tumor infiltrating lymphocyte
TISIDB: Tumor and Immune System Interaction Database
TME: the tumor microenvironment
Treg: regulatory T
UCEC: uterine corpus endometrial carcinoma
UCS: uterine carcinosarcoma
UVM: uveal melanoma

Dear Editor,

Immunotherapy has become a major form of cancer therapy after chemotherapy, radiotherapy, and targeted therapy. Immune checkpoint inhibitors (ICIs), such as anti‐programmed cell death 1 (PD1), anti‐programmed cell death ligand 1 (PDL1), and anti‐cytotoxic T‐lymphocyte associated protein 4 (CTLA4), are widely studied in cancer immunotherapy.^1^, ^2^ However, a considerable percentage of tumor patients fail to respond to ICI monotherapy.^3^ Researchers have focused on enhancing mono‐ICI efficacy through combination therapy or exploring novel immunotherapy targets. Lymphocyte‐associated gene 3 (LAG3), a promising immune checkpoint, has received increasing attention recently. In this study, we described the biology of LAG3 and its function in cancer immunology, explored the multiomics characteristics of LAG3 utilizing a bioinformatics database, and provided perspective on the applications of single therapy or potential combination strategies for LAG3‐targeting agents.

LAG3 is a type I transmembrane protein that can be cut by metalloproteinase to release soluble LAG3 (sLAG3). LAG3 is expressed on CD4+, CD8+, regulatory T (Treg) cell, natural killer cell, B cell, and other immune cells.^4^ LAG3 has been reported to play a negative regulatory role in cancer immunology by interacting with its ligands, including major histocompatibility complex class II (MHC II), galectin‐3, liver sinusoidal endothelial cell lectin, and fibrinogen‐like protein 1^5^, ^6^ (Figure 1A). For example, the LAG3–MHC II interaction can downregulate T cell proliferation and protect melanoma cells from drug‐induced apoptosis.^7^, ^8^ sLAG3 expression was positively correlated with dendritic cell migration and T cell antitumor ability.^9^

**FIGURE 1:**
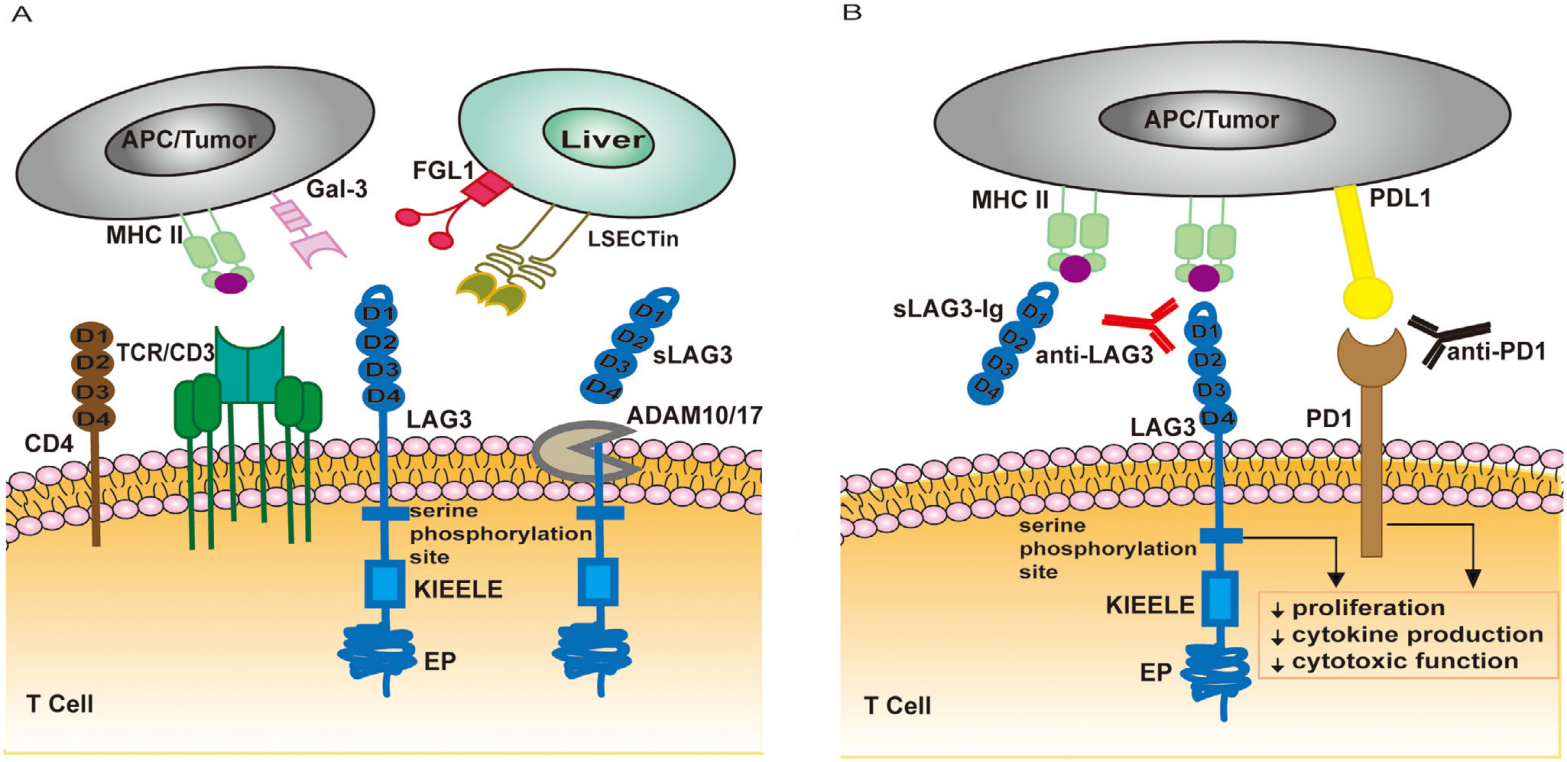
LAG3 biology and the mechanisms of LAG3‐targeting agents. (A) LAG3 structure and its ligands. Like CD4, LAG3 consists of an extracellular, transmembrane, and an intracellular region. The interaction of LAG3 and MHC II interferes with the binding of the MHC II to CD4. LAG3 has also been reported to bind to Gal‐3, LSECtin, and FGL1. LAG3 downregulates effector cell proliferation, cytokine production, and cytotoxicity by binding to its ligands. (B) The mechanisms of targeting LAG3. sLAG3 can activate APC and restore T cell function. LAG3 mAb blocks the inhibitory pathways between LAG3 and its ligands to release immune brakes. Abbreviations: APC, antigen‐presenting cell; FGL1, fibrinogen‐like protein 1; Gal‐3, galectin‐3; LAG3, lymphocyte‐associated gene 3; LSECtin, liver sinusoidal endothelial cell lectin; MHC II, major histocompatibility complex II; sLAG3, soluble LAG3

To better guide the application of LAG3‐targeting agents in cancer immunotherapy, we explored the immunomodulatory role of LAG3 in the tumor microenvironment (TME). We utilized the Gene Expression Profiling Interactive Analysis (GEPIA, http://gepia2.cancer‐pku.cn) database to analyze the expression of LAG3 and other common immune checkpoints across 33 cancers (Figure 2A). The expression of *lag3* in kidney renal clear cell carcinoma (KIRC), pancreatic adenocarcinoma (PAAD), skin cutaneous melanoma (SKCM), testicular germ cell tumors (TGCT), lymphoid neoplasm diffuse large B‐cell lymphoma (DLBC), and head and neck squamous cell carcinoma (HNSC) was significantly higher than in paired normal tissues, suggesting that blocking LAG3 may have a remarkable antitumor effect in these cancers. The expressions of *lag3* and *pdcd1* in TGCT, *lag3* and *ctla4* in HNSC and PAAD, *lag3*, *pdcd1*, and *ctla4* in SKCM, and *lag3*, *pdcd1*, *cd274*, and *ctla4* in DLBC were significantly higher than in paired normal tissues. This provides a theoretical basis for LAG3‐targeting agents in combination with other common ICIs.

**FIGURE 2:**
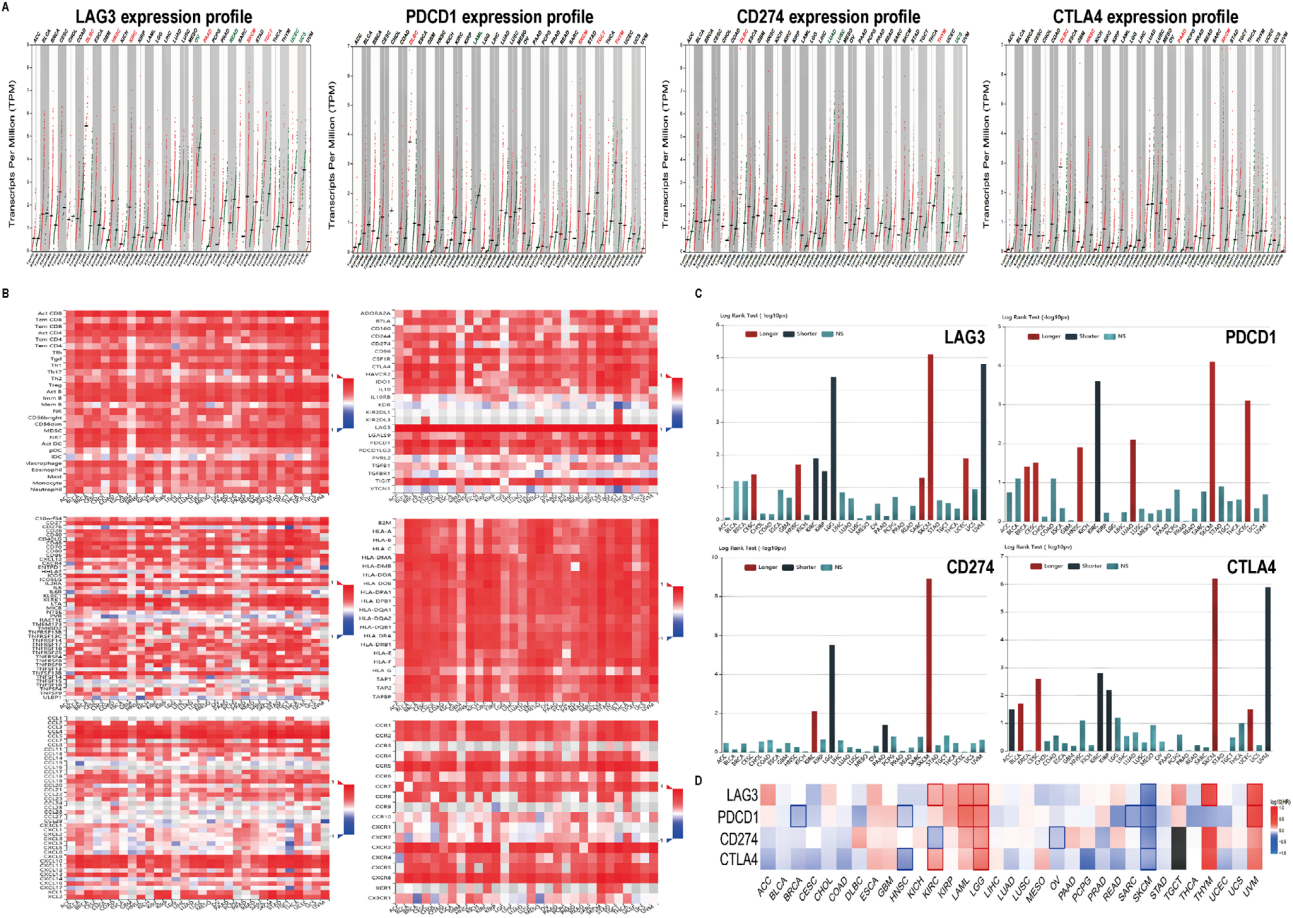
Multiomics analysis of LAG3 and other common immune checkpoints in a Pan‐cancer analysis. (A) *Lag3* and *pdcd1* / *cd274* / *ctla4* expression profiles across all tumor samples and paired normal tissues. Each dot represents a distinct tumor or normal sample. Red text of each cancer type indicates that the gene is overexpressed in tumors than in normal tissues. Green text of each cancer type indicates that the gene is underexpressed in tumors than in normal tissues. Four‐way analysis of variance, using sex, age, ethnicity, and disease state (tumor or normal) as variables was applied to calculate differential expression. The expression data were log_2_ (TPM + 1) transformed. *p* < 0.01 was considered statistically significant. (B) Spearman's correlation of *lag3* with immune features across multiple cancers. *p* < 0.05 was considered statistically significant. (C) Association analyses between *lag3* / *pdcd1* / *cd274* / *ctla4* and clinical prognosis across multiple cancers. Red bars signify that high levels of the molecule are significantly associated with longer OS. Blue bars signify that high levels of the molecule are significantly associated with decreased OS. A log rank test was used to calculate the associations. (D) The survival heat map shows the prognostic impact of *lag3* / *pdcd1* / *cd274* / *ctla4*. With an increase in gene expression, the red and blue blocks denote high and low risks, respectively. The rectangles with frames indicate the significant unfavorable and favorable results in prognostic analyses. Mantel–Cox test was used to compare the survival contribution of these genes. *p* < 0.05 was considered statistically significant. Abbreviations: CTLA4, cytotoxic T‐lymphocyte associated protein 4; LAG3, lymphocyte‐associated gene 3; PDCD1, programmed cell death 1

We utilized the Tumor and Immune System Interaction Database (TISIDB) (http://cis.Hku.hk/TISIDB) to show the correlation between *lag3* expression and tumor infiltrating lymphocyte (TIL) abundance, immunoregulatory factors, and chemokines across 30 cancer types (Figure 2B). The results showed that (1) *lag3* expression was correlated with the abundance of multiple TILs, such as activated CD8+ T cell, Treg cell, and myeloid‐derived suppressor cell, which supports the dual negative regulatory role of LAG3 in TME. LAG3 downregulates the antitumor efficacy of effector cell and enhances the inhibitory effect of suppressor cell.^10^ (2) *Lag3* expression was positively associated with other immune checkpoints, such as *pdcd1* and *ctla4* in multiple cancers. Similar conclusions can be drawn from the GEPIA database (Figure 3). *Lag3* expression was highly correlated with *pdcd1* in SKCM and kidney renal papillary cell carcinoma (KIRP), suggesting that LAG3 and PD1 cotargeted immunotherapy may induce strong synergistic antitumor properties in both cancers. (3) *Lag3* expression was positively correlated with many immunostimulators, such as *cd80* and *cd86*, suggesting that LAG3 regulates immune homeostasis together with immunostimulators. (4) *Lag3* expression was positively correlated with almost all MHC‐related genes, suggesting that LAG3 may interact with MHC molecules, other than MHC II. (5) *Lag3* expression was positively correlated with reported chemokines and chemokine receptors, such as *cxcl2*, *cxcl5*, and *ccr2*. Therefore, the relationship between *lag3* and chemokines needs to be further investigated.

**FIGURE 3:**
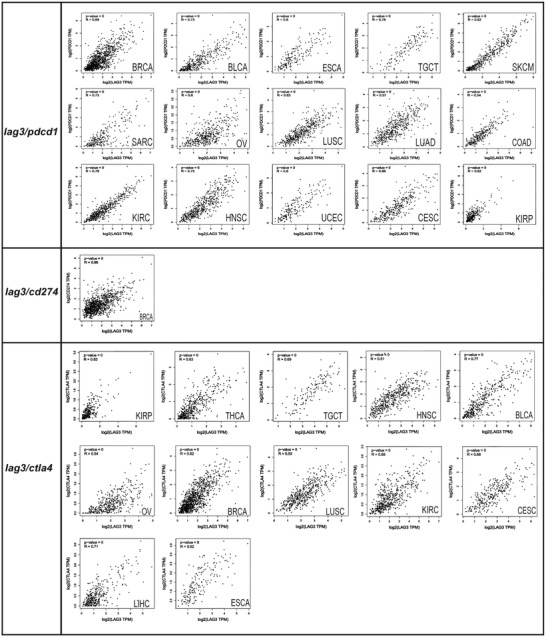
Correlation analysis between *lag3* and *pdcd1* / *cd274* / *ctla4* across multiple cancers. Each dot represents a distinct tumor or normal sample. The non‐log scale was used for calculation and the log‐scale axis was used for visualization. Pearson test was used to analyze the correlation between *lag3* and *pdcd1* / *cd274* / *ctla4*. *p* < 0.05 was considered statistically significant

Further correlations between *lag3*, *pdcd1*, *cd274*, and *ctla4* expression and the clinical prognosis across 30 cancer types were analyzed utilizing TISIDB (Figure 2C). The high expression of *lag3* was negatively correlated with the overall survival (OS) of KIRC, KIRP, brain lower grade glioma (LGG), and uveal melanoma (UVM) patients, suggesting that *lag3* plays a pivotal role in promoting tumor growth in these tumors. The following high expressions were all negatively correlated: *pdcd1* and the OS of KIRC patients, *cd274* and the OS of LGG and PAAD patients, *ctla4* and the OS of adrenocortical carcinoma, KIRC, KIRP, and UVM patients. We obtained similar results from the GEPIA database (Figure 2D). Interestingly, *lag3* expression was positively correlated with the OS of patients with several types of tumors (Figure 2C), which is contradictory to the inhibitory role of LAG3 in the immune system. It may be due to the complicated tumor environment and different clinical features, such as the disease stage, initial treatments, and other heterogeneous factors in databases, that deserve further exploration.

There are two types of LAG3‐targeting agents used as antitumor immunotherapies: LAG3 soluble dimeric recombinant protein named IMP321 and LAG3 mAb (Figure 1B). IMP321 acts as an antigen‐presenting cell activator to exert an antitumor effect. LAG3 mAb blocks the binding of LAG3 and its ligands to improve the antitumor activity of the host, which is widely applied in drug discovery. Dozens of IMP321 and LAG3 mAb‐related clinical trials for various cancers are currently underway, most of which are combined with anti‐PD1 (Table 1). LAG3 and PD1 / PDL1 / CTLA4 bispecific antibody immunotherapy is also in progress (Table S1).

**TABLE 1:** Clinical studies of LAG3 single‐targeted immunotherapy

Drugs	NCT ID	Tumor types	Phase	Number enrolled	Combination agents (targeting LAG3 drugs + X)	Status
**Soluble LAG3 Ig**						
IMP321	NCT03252938	Solid tumors	I	50	Avelumab	Recruiting
	NCT03625323	NSCLC, SCCHN	II	109	Pembrolizumab	Recruiting
	NCT00351949	Advanced RCC	I	24	–	Completed
	NCT02676869	Stage III/IV melanoma	I	24	Pembrolizumab	Completed
	NCT02614833	Adenocarcinoma breast stage IV	II	241	Paclitaxel	Active, not recruiting
	NCT00349934	Metastatic breast cancer	I	33	Paclitaxel	Completed
**Anti‐LAG3 mAb**						
BMS986016 Relatlimab	NCT02966548	Advanced solid tumors	I	45	Nivolumab	Active, not recruiting
	NCT02061761	Hematologic neoplasms	I/II	109	Nivolumab	Active, not recruiting
	NCT03743766	Melanoma	II	42	Nivolumab	Recruiting
	NCT01968109	Neoplasms by site	I/II	1500	Nivolumab, BMS‐986213	Recruiting
	NCT03623854	Chordoma	II	20	Nivolumab	Recruiting
	NCT03493932	Glioblastoma	I	25	Nivolumab	Recruiting
	NCT03642067	Colorectal adenocarcinoma	II	64	Nivolumab	Recruiting
	NCT03459222	Advanced cancer	I/II	230	Nivolumab, Ipilimumab, BMS986205	Recruiting
	NCT04326257	SCCHN	II	40	Nivolumab, Ipilimumab	Recruiting
	NCT03607890	Refractory MSI ‐ H solid tumors prior of PD‐L1 therapy	II	21	Nivolumab	Recruiting
	NCT02488759	Advanced cancer	I/II	584	Nivolumab, Ipilimumab, Daratumumab	Active, not recruiting
	NCT02658981	Gliosarcoma	I	100	Nivolumab	Recruiting
	NCT02996110	Advanced cancer	II	200	Nivolumab, Ipilimumab	Recruiting
	NCT02750514	Advanced cancer	II	504	Dasatinib, Nivolumab	Active, not recruiting
	NCT02060188	Microsatellite unstable colorectal cancer	II	340	Nivolumab, Ipilimumab, Cobimetinib	Recruiting
	NCT04080804	SCCHN	II	60	Nivolumab, Ipilimumab	Recruiting
	NCT02935634	Advanced gastric cancer	II	600	Nivolumab, Ipilimumab	Recruiting
	NCT02519322	Cutaneous melanoma	II	53	Nivolumab, Ipilimumab	Recruiting
	NCT03044613	Gastric cancer	I	25	Carboplatin, Nivolumab	Recruiting
	NCT04062656	Gastric cancer	II	88	Nivolumab, Ipilimumab	Recruiting
	NCT03335540	Advanced cancer	I	50	Cabiralizumab, Nivolumab	Recruiting
LAG525	NCT03499899	Triple negative breast cancer	II	88	Spartalizumab	Active, not recruiting
	NCT03365791	Advanced solid and hematologic malignancies	II	76	PDR001	Active, not recruiting

Abbreviations: LAG3, lymphocyte‐associated gene 3; NSCLC, non‐small cell lung cancer; PDL1, programmed cell death ligand 1; RCC, renal cell carcinoma; SCCHN, head and neck squamous cell carcinoma.

ICIs have greatly benefited tumor patients, and combination therapy improved the efficacy of ICIs. LAG3 is a promising checkpoint that negatively regulates T cell activation and indicates a poor prognosis for KIRC, KIRP, and many other tumors. The single application of LAG3‐targeting agents in the dominant population and in combination with other ICIs, such as anti‐PD1 / PDL1 / CTLA4, is expected to benefit more tumor patients. We hope that more clinical trials of LAG3‐targeting agents in combination with chemotherapy, radiotherapy, and targeted therapy could be performed to obtain encouraging results.

## COMPETING INTERESTS

The authors declare that they have no competing interests.

## AUTHORS CONTRIBUTION

Q.L. contributed to the design of the review and revised the article, had full access to all the contents included in this study, and took responsibility for the integrity of the data and the accuracy of the data analysis. M.W. and J.J. collected the literature. M.W. performed the bioinformatics analysis, prepared the figures, and performed the data interpretation. M.W., Q.D., Y.W., and Y.L. wrote the main manuscript text. All the authors contributed to the review and revision of the manuscript, and all authors read and approved the final manuscript.

## AVAILABILITY OF DATA AND MATERIALS

All data generated or analyzed during this study are included.

## CONSENT FOR PUBLICATION

All authors approved the final manuscript for publication.

## FUNDING

This work was supported by the Research Foundation of Beijing Friendship Hospital, Capital Medical University (No. yyqdky2019‐40 and No. yyzscq202003).

## Supporting information


**Table S1** Clinical studies of LAG3 and other immune checkpoints cotargeted immunotherapy
