# Supplementary material for: LAG3 and its emerging role in cancer immunotherapy
Source: Clin Transl Med. 2021 Mar 24;11(3):e365. doi: 10.1002/ctm2.365 (PMC7989707; doi:10.1002/ctm2.365)
Supplement: Supplementary file 1 — Table S1 Clinical studies of LAG3 and other immune checkpoints cotargeted immunotherapy [file CTM2-11-e365-s001.docx]

**Table S1** Clinical Studies of LAG3 and other immune checkpoints co-targeted immunotherapy

| Drugs | NCT ID | Tumor types | Phase | Number Enrolled | Combination agents  (Targeting LAG3 drugs + X) | Status |
| --- | --- | --- | --- | --- | --- | --- |
| **LAG3/PD1 bispecific DART antibody** | | | | | | |
| MGD013 | NCT04212221 | Advanced Hepatocellular Carcinoma | I/II | 300 | Brivanib, Alaninate | Recruiting |
|  | NCT03219268 | Advanced Solid Tumors,  Hematologic Neoplasms | I | 375 | margetuximab | Recruiting |
|  | NCT04178460 | Gastric Cancer | I | 59 | Niraparib | Recruiting |
|  | NCT04082364 | Gastric Cancer | II/III | 850 | margetuximab | Recruiting |
| RO-7247669 | NCT04140500 | Solid Tumors | I | 200 | -- | Recruiting |
| **LAG3/PDL1 bispecific antibody** | | | | | | |
| FS118 | NCT03440437 | Advanced Cancer | I | 43 | -- | Active, not recruiting |
| **LAG3/CTLA4 bispecific antibody** | | | | | | |
| Xmab-22841 | NCT03849469 | Advanced Solid Tumors | I | 242 | Pembrolizumab | Recruiting |

Abbreviations: LAG3: Lymphocyte-associated gene 3; PD1: Programmed cell death 1; PDL1: Programmed cell death ligand 1; CTLA4: Cytotoxic T-lymphocyte associated protein 4; mAb: Monoclonal antibody; DART: Dual-affinity re-targeting.
